# Exploring *Mycobacterium riyadhense*: Epidemiology, Clinical Presentation, and Treatment Outcome

**DOI:** 10.1093/ofid/ofaf461

**Published:** 2025-08-01

**Authors:** Mohammed Alsaeed, Khalid Alanazi, Ali Alhamdan, Mohammed Faqihi, Alaa Alibrahim, Shahad Alshehri, Diaa Shosha, Mohammed Alraddadi, Mikqdad Alsaeed, Mohammed Alabdullah, Sirine Ahmad

**Affiliations:** Medicine Department, Infectious Diseases Division, Prince Sultan Military Medical City, Ministry of Defence Health Services General Directorate, Riyadh, Saudi Arabia; Medicine Department, Dr. Sulaiman Al Habib Medical Group, Riyadh, Saudi Arabia; College of Medicine, Alfaisal University, Riyadh, Saudi Arabia; Medicine Department, Infectious Diseases Division, Prince Sultan Military Medical City, Ministry of Defence Health Services General Directorate, Riyadh, Saudi Arabia; Department of Adult Infectious Diseases, King Saud Medical City, Riyadh, Saudi Arabia; Microbiology Division, Pathology and Laboratory Medicine, Prince Sultan Military Medical City, Riyadh, Saudi Arabia; Medicine Department, Infectious Diseases, College of Medicine, Jouf University, Sakaka, Saudi Arabia; Medicine Department, Infectious Diseases Unit, Prince Mohammed bin Abdulaziz Hospital, Riyadh, Saudi Arabia; Medicine Department, Infectious Diseases Unit, Prince Mohammed bin Abdulaziz Hospital, Riyadh, Saudi Arabia; Medicine Department, Infectious Diseases Division, Prince Sultan Military Medical City, Ministry of Defence Health Services General Directorate, Riyadh, Saudi Arabia; Department of Internal Medicine, King Saud Medical City, Riyadh, Saudi Arabia; Infectious Diseases Department, Almoosa Specialist Hospital, Al Mubarraz, Saudi Arabia; Medicine Department, Dr. Sulaiman Al Habib Medical Group, Riyadh, Saudi Arabia

**Keywords:** *Mycobacterium riyadhense*, nontuberculous mycobacteria

## Abstract

**Background:**

*Mycobacterium riyadhense*, an emerging nontuberculous mycobacterium (NTM), closely resembles *Mycobacterium tuberculosis* (TB) clinically, often complicating its diagnosis and management.

**Methods:**

We retrospectively analyzed 8 new cases of *M riyadhense* infection diagnosed at Prince Sultan Military Medical City from 2019 to 2024. Additionally, a systematic review was conducted of 24 previously reported cases from 2009 to 2025, identified through extensive searches of PubMed and Google Scholar databases. Data extracted included patient demographics, clinical features, diagnostic methods, treatments administered, and clinical outcomes.

**Results:**

Pulmonary infections were predominant and frequently mistaken for TB, resulting in diagnostic delays. Extrapulmonary infections included lymphadenitis and osteomyelitis. A novel association with immune complex glomerulonephritis was identified. Molecular sequencing was critical in confirming diagnoses due to limitations in conventional microbiological techniques. Treatment regimens based on macrolides and fluoroquinolones yielded superior therapeutic outcomes, exhibiting lower relapse rates and fewer adverse effects compared with conventional anti-TB therapy. Surgical interventions played a crucial role in managing complicated or refractory cases.

**Conclusions:**

Enhancing clinical awareness, employing accurate molecular diagnostic techniques, and adopting targeted antimicrobial therapy are essential for effective management of *M riyadhense* infections. Further research is needed to optimize treatment protocols and improve patient outcomes.

*Mycobacterium riyadhense*, first described in 2009 in Riyadh, Saudi Arabia, is a nontuberculous mycobacterium (NTM) initially misidentified as part of the *Mycobacterium tuberculosis* complex (MTBC) by commercial line-probe assays [1]. Its clinical significance has since been reported in France, Bahrain, South Korea, and the UAE, with presentations mimicking pulmonary tuberculosis (TB) and, less commonly, extrapulmonary infections such as lymphadenitis and osteomyelitis [2–7]. *M riyadhense* shares clinical and immunological features with both *M tuberculosis* and other NTMs, such as *Mycobacterium avium* complex (MAC), particularly in its pulmonary manifestations and response to macrolide-based therapy, though it often resembles TB radiologically [11]. Definitive diagnosis requires molecular techniques (eg, 16S rRNA and hsp65 gene sequencing) to distinguish it from MTBC and other NTMs [3, 7]. Treatment often follows TB or NTM guidelines due to the lack of standardized protocols [8, 9]. To enhance understanding of this emerging pathogen, we analyzed 8 new cases diagnosed at Prince Sultan Military Medical City (2019–2024) and systematically reviewed 24 previously reported cases (2009–2025).

## METHODS

We conducted a retrospective analysis of eight *M riyadhense* cases diagnosed at Prince Sultan Military Medical City, Riyadh, from 2019 to 2024. Cases were identified through microbiology records, with diagnosis confirmed by culture and molecular sequencing (16S rRNA, hsp65). Data extracted included patient demographics, comorbidities, clinical presentation, radiological findings, diagnostic methods, treatment regimens, and outcomes. A systematic review of 24 previously reported cases was performed using PubMed and Google Scholar (2009–2025), with search terms including “*Mycobacterium riyadhense*,” “nontuberculous mycobacteria,” and “NTM infection.” The study was approved by the Institutional Review Board of Prince Sultan Military Medical City.

## RESULTS

### Epidemiology

Among 32 total cases (8 new, 24 prior), 22 (68.75%) were reported in Saudi Arabia, predominantly in Riyadh, supporting regional endemicity (Tables 1, 2) [7]. Additional cases from France (1 case), Bahrain (1 case), South Korea (1 case), and the UAE (1 case) suggest global dissemination, likely facilitated by travel or migration (Table 1) [2, 3, 9]. The age range was 8–82 years (median 39 years), with a balanced gender distribution (17 males, 15 females). Host factors varied: 20 cases (62.5%) were immunocompetent, while 12 (37.5%) were immunosuppressed, including 6 with HIV, 1 with systemic lupus erythematosus (SLE), and 1 with advanced retroviral disease (Tables 1 and 2). Patients who are immunosuppressed were younger (median 37 years vs 44 years for immunocompetent; *P* = .04). No consistent environmental exposures (eg, water sources and soil) or person-to-person transmission were identified, though 3 cases reported recent surgery (eg, gastric sleeve and gastrectomy), suggesting possible healthcare-associated acquisition (Table 2; cases 1 and 8). The regional clustering in Riyadh underscores the need to investigate local environmental reservoirs.

**Table 1.** Summary of Previously Reported *M riyadhense* Cases (2009–2025)

Case (Citation)	Year	Region	Age/Gender	Comorbidities	Site	Symptoms	Radiology	Diagnostic Method	Initial Regimen	Modified Regimen	Duration	Outcome	Delay (Weeks)	Notes	Reference
1 [1]	2009	KSA	19/M	None	Maxillary sinus	Sinus pain	Sinus opacity	Culture, 16S rRNA	INH, RIF, EMB, PZA	None	9 months	Cured	6	-	[1]
2 [2]	2005	France	39/F	None	Pulmonary	Cough, fever	Cavitary lesions	Culture, hsp65	INH, RIF, EMB, PZA	INH, RIF	12 months	Cured	5	-	[2]
3 [2]	2006	Bahrain	43/M	None	Pulmonary	Cough, dyspnea	Consolidation	Culture, 16S rRNA	CLR, CIP	INH, RIF, EMB, PZA, CLR, CIP	20 months	Cured	7	Relapse	[2]
4 [3]	2011	Korea	38/F	None	Pulmonary	Cough, fever	Cavitary lesions	Culture, hsp65	INH, RIF, EMB, PZA	RIF, EMB, PZA	13 months	Cured	6	-	[3]
5 [4]	2013	KSA	54/M	HIV	Pulmonary	Cough, weight loss	Nodules	Culture, 16S rRNA	INH, RIF, EMB, CLR	RIF, EMB, CLR	12 months	Cured	8	-	[4]
6 [5]	2012	KSA	18/F	None	Cranium	Headache	Osteolytic lesions	Biopsy, 16S rRNA	INH, RIF, EMB, MFX	RIF, EMB	15 m	Cured	5	-	[5]
7 [5]	2012	KSA	24/F	None	Spine	Back pain	Vertebral lesions	Biopsy, hsp65	INH, RIF, EMB, PZA	RIF, EMB	13 m	Cured	6	-	[5]
8 [6]	2016	KSA	30/M	HIV	Pulmonary	Cough, fever	Consolidation	Culture, 16S rRNA	INH, EMB, PZA, MFX	None	N/R	Cured	7	-	[6]
9 [7]	2014	KSA	25/M	None	Pulmonary	Cough	Cavitary lesions	Culture, 16S rRNA	INH, RIF, CLR	N/R	N/R	Cured	6	-	[7]
10 [7]	2014	KSA	55/M	None	Pulmonary	Cough, dyspnea	Consolidation	Culture, 16S rRNA	INH, RIF, EMB, PZA	N/R	N/R	Cured	5	-	[7]
11 [7]	2014	KSA	39/F	None	Pulmonary	Cough, fever	Nodules	Culture, hsp65	INH, RIF, EMB, PZA	N/R	N/R	Cured	6	-	[7]
12 [7]	2014	KSA	77/M	None	Pulmonary	Cough	Cavitary lesions	Culture, 16S rRNA	INH, RIF	N/R	N/R	Cured	5	-	[7]
13 [7]	2014	KSA	57/M	None	Lymph nodes	Neck swelling	Lymphadenopathy	Biopsy, 16S rRNA	INH, RIF, CLR	N/R	N/R	Cured	7	-	[7]
14 [7]	2014	KSA	82/M	None	Pulmonary	Cough, weight loss	Consolidation	Culture, 16S rRNA	INH, RIF, CLR	N/R	N/R	Cured	6	-	[7]
15 [7]	2014	KSA	18/M	None	Pulmonary	Cough, fever	Cavitary lesions	Culture, hsp65	INH, RIF, EMB, PZA	N/R	N/R	Cured	5	-	[7]
16 [7]	2014	KSA	32/M	None	Pulmonary	Cough	Nodules	Culture, 16S rRNA	INH, RIF, CLR	N/R	N/R	Cured	6	-	[7]
17 [7]	2014	KSA	61/M	None	Pulmonary	Cough, dyspnea	Consolidation	Culture, 16S rRNA	INH, RIF	N/R	N/R	N/A	5	-	[7]
18 [7]	2014	KSA	8/M	None	Lymph nodes	Neck swelling	Lymphadenopathy	Biopsy, 16S rRNA	INH, RIF, CLR	N/R	N/R	Cured	7	-	[7]
19 [7]	2014	KSA	82/M	None	Pulmonary	Cough, weight loss	Cavitary lesions	Culture, hsp65	INH, RIF	N/R	N/R	Died	6	-	[7]
20 [7]	2014	KSA	28/M	None	Lymph nodes	Neck swelling	Lymphadenopathy	Biopsy, 16S rRNA	INH, RIF	N/R	N/R	Cured	7	-	[7]
21 [8]	2013	KSA	44/F	HIV	Pulmonary	Cough, fever	Nodules	Culture, 16S rRNA	AZM, EMB, MFX	None	12 months	Cured	8	-	[8]
22 [8]	2015	KSA	51/M	HIV	Pulmonary	Cough, weight loss	Consolidation	Culture, hsp65	MFX, CLR	None	10 months	Cured	7	-	[8]
23 [9]	2021	UAE	44/F	None	Pulmonary	Cough, dyspnea	Cavitary lesions	Culture, 16S rRNA	INH, RIF, EMB, PZA	INH, RIF, EMB, PZA, CLR	Ongoing	Improved	6	-	[9]
24 [13]	2022	KSA	39/F	None	Cranium/sternum	Chest pain	Osteolytic lesions	Biopsy, 16S rRNA	INH, RIF, EMB, CLR, MFX	INH, RIF, EMB	12 months	Cured	5	-	[13]

Abbreviations: M, male; F, female; HIV, human immunodeficiency virus; INH, isoniazid; RIF, rifampicin; EMB, ethambutol; PZA, pyrazinamide; CLR, clarithromycin; CIP, ciprofloxacin; MFX, moxifloxacin; AZM, azithromycin; KSA, Saudi Arabia; N/R, not reported; N/A, not applicable.

**Table 2.** Summary of New *M Riyadhense* Cases

Case	Year	Age/Gender	Comorbidities	Site	Symptoms	Radiology	Diagnostic Method	Initial Regimen	Modified Regimen	Duration	Outcome	Delay (Weeks)	Notes
1	2019	19/F	Morbid obesity	Lymph nodes	Neck swelling, pus	Necrotic lymph nodes (Figure 1)	Culture, 16S rRNA	RIF, EMB, CLR	RIF, MFX	12 months	Cured	7	Postgastric sleeve
2	2019	47/M	HIV, CAD	Pulmonary	Cough, fever, weight loss	Tree-in-bud, nodules (Figure 2)	Culture, 16S rRNA	RFB, EMB, CLR	EMB, CLR	11 months	Cured	8	CMV retinitis
3	2021	36/F	SLE, APS	Pulmonary	Cough	Cavitary lesions (Figure 3)	BAL, hsp65	INH, RIF, EMB, PZA	INH, EMB, MFX	13 m	Cured	5	Hepatotoxicity
4	2022	40/M	Smoker	Pulmonary	Cough, dyspnea, fever	Consolidation, cavitation (Figure 4)	Biopsy, 16S rRNA	INH, RIF, EMB, PZA	RIF, EMB, CLR	9 months	Cured	8	Initial refusal
5	2022	28/F	None	Pulmonary	Cough	Cavitary lesions, consolidation (Figure 5)	Culture, 16S rRNA	RIF, EMB, CLR	None	12 months	Cured	7	-
6	2023	55/M	HIV, smoker	Pulmonary	Cough, hemoptysis	Subpleural lesion (Figure 6)	EBUS, hsp65	INH, RIF, EMB, AZM	None	N/R	Improved	8	Cancer mimic
7	2023	37/M	HIV, epilepsy	Osteomyelitis	Foot pain, swelling	Metatarsal lesion (Figure 7)	Biopsy, 16S rRNA	INH, RIF, EMB, AZM	RIF, EMB, MFX	15 months	Cured	5	IRIS
8	2024	24/M	Gastrectomy	Pulmonary, GN	Cough, fever, anemia	Cavitary lesions (Figure 8)	BAL, hsp65	INH, RIF, EMB, PZA	RIF, EMB, AZM	Ongoing	Improved	6	Glomerulonephritis

Abbreviations: F, female; M, male; HIV, human immunodeficiency virus; CAD, coronary artery disease; SLE, systemic lupus erythematosus; APS, antiphospholipid syndrome; GN, glomerulonephritis; RIF, rifampicin; RFB, rifabutin; EMB, ethambutol; PZA, pyrazinamide; CLR, clarithromycin; MFX, moxifloxacin; AZM, azithromycin; BAL, bronchoalveolar lavage; EBUS, endobronchial ultrasound; IRIS, immune reconstitution inflammatory syndrome; N/R, not reported.

### Clinical Presentation

**Figure 1.**
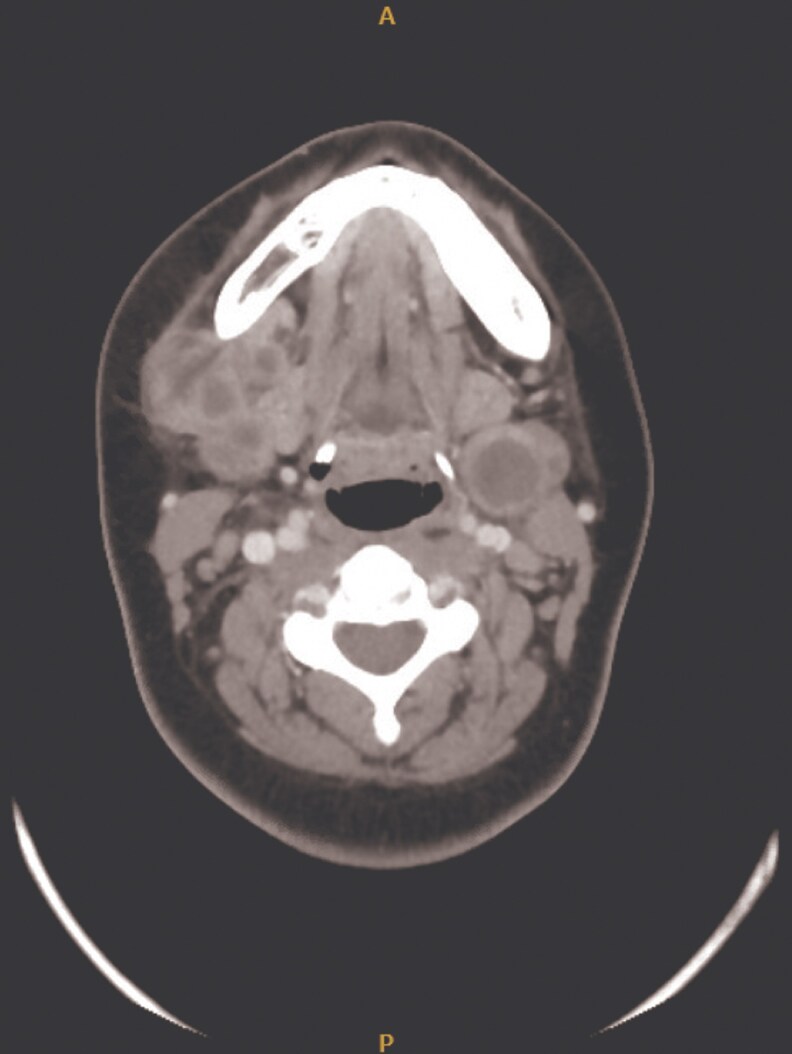
Imaging or clinical photograph depicting necrotic lymph nodes associated with lymphadenitis in a case of *M riyadhense* infection (case 1; Table 2).

**Figure 2.**
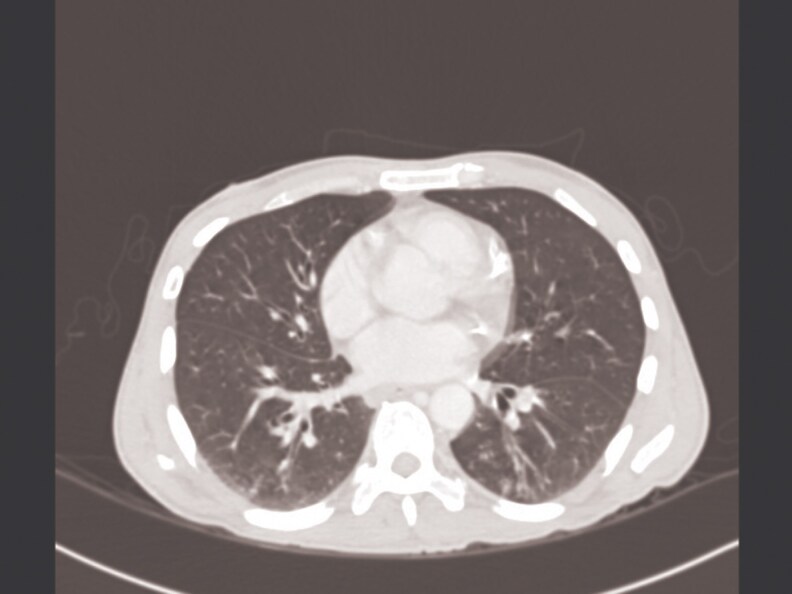
Radiological image (eg, CT scan) showing tree-in-bud pattern and nodules in a pulmonary *M riyadhense* infection (case 2; Table 2).

**Figure 3.**
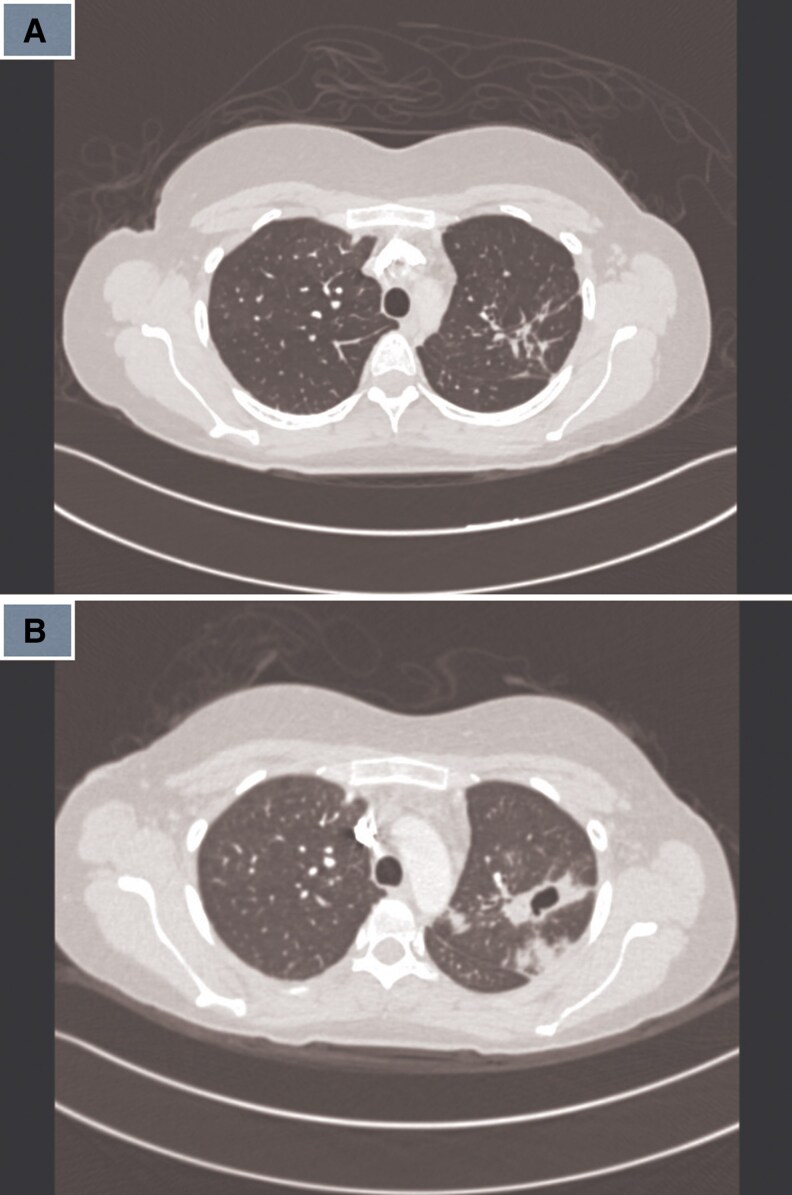
(*A*) Radiological image (eg, CT scan) showing cavitary lesions in a pulmonary *M riyadhense* infection (case 3; Table 2). (*B*) Follow-up imaging showing resolution of cavitary lesions posttreatment (case 3; Table 2).

**Figure 4.**
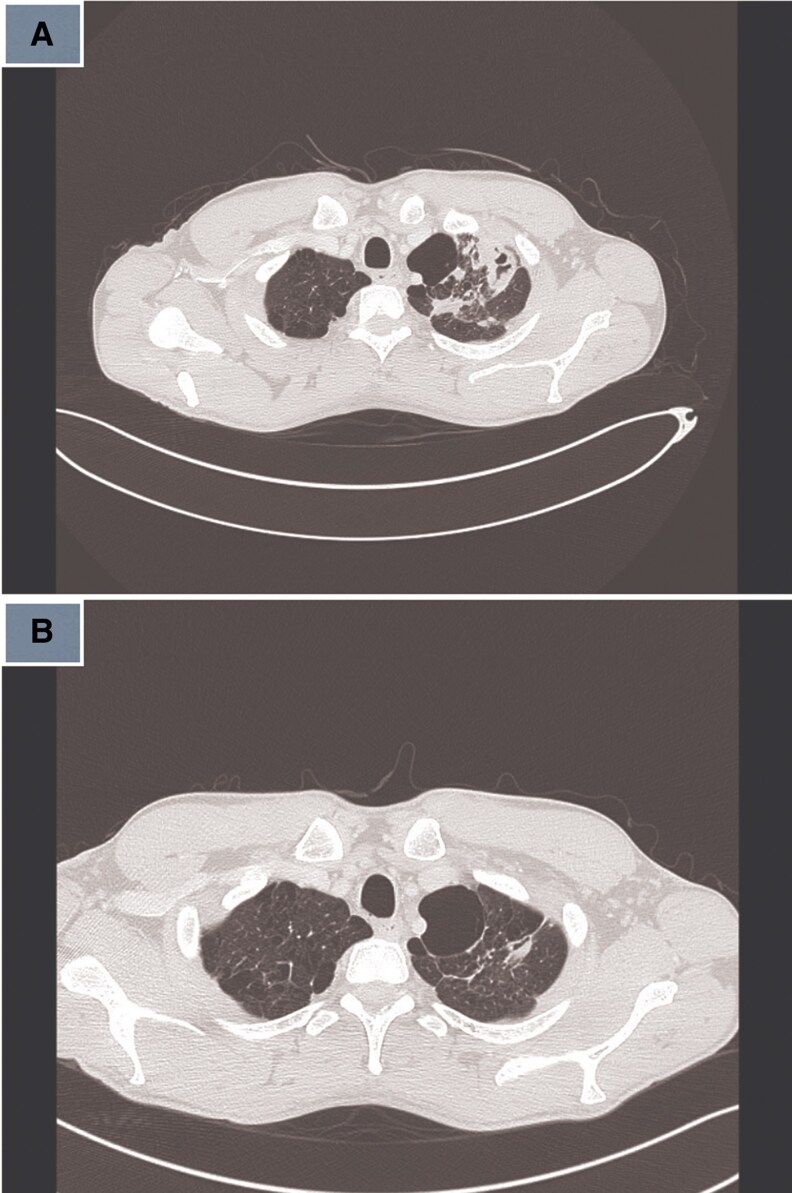
(*A*) Radiological image (eg, CT scan) showing consolidation and cavitation in a pulmonary *M riyadhense* infection (case 4; Table 2). (*B*) Follow-up imaging showing resolution of lesions posttreatment (case 4; Table 2).

**Figure 5.**
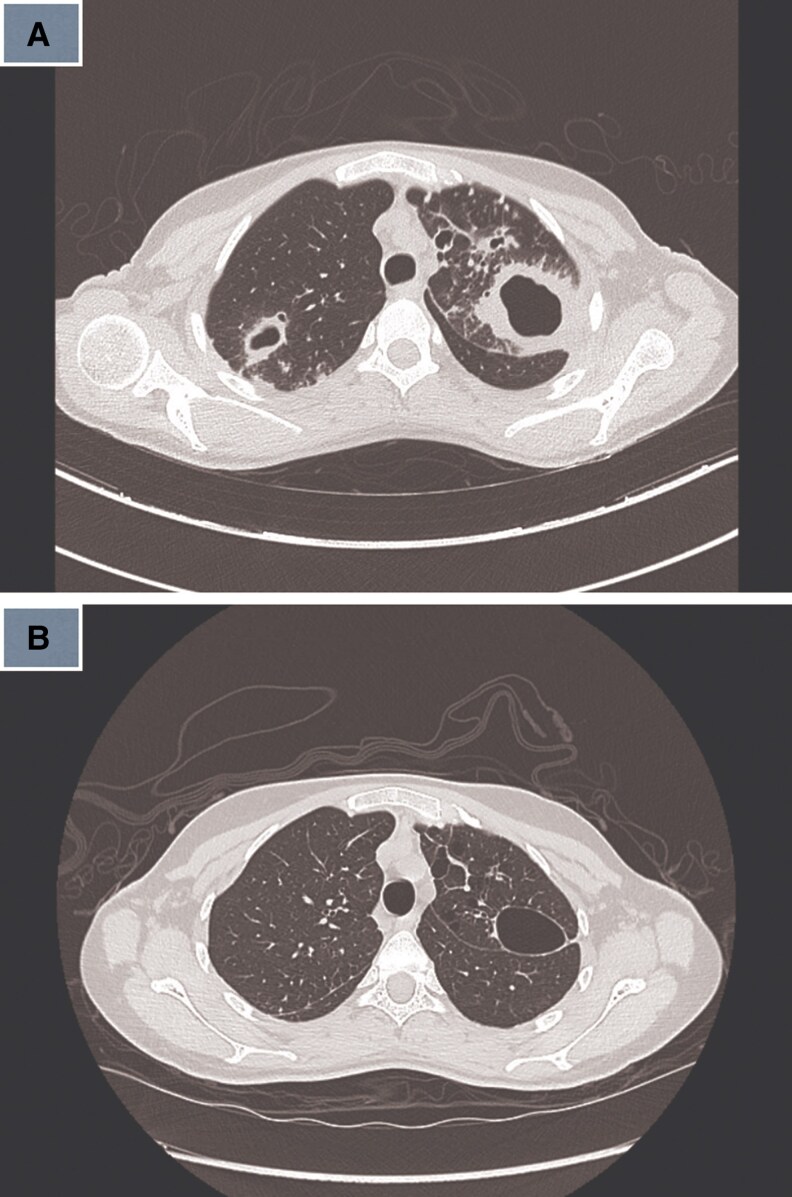
(*A*) Radiological image (eg, CT scan) showing cavitary lesions and consolidation in a pulmonary *M riyadhense* infection (case 5; Table 2). (*B*) Follow-up imaging showing resolution of lesions posttreatment (case 5; Table 2).

**Figure 6.**
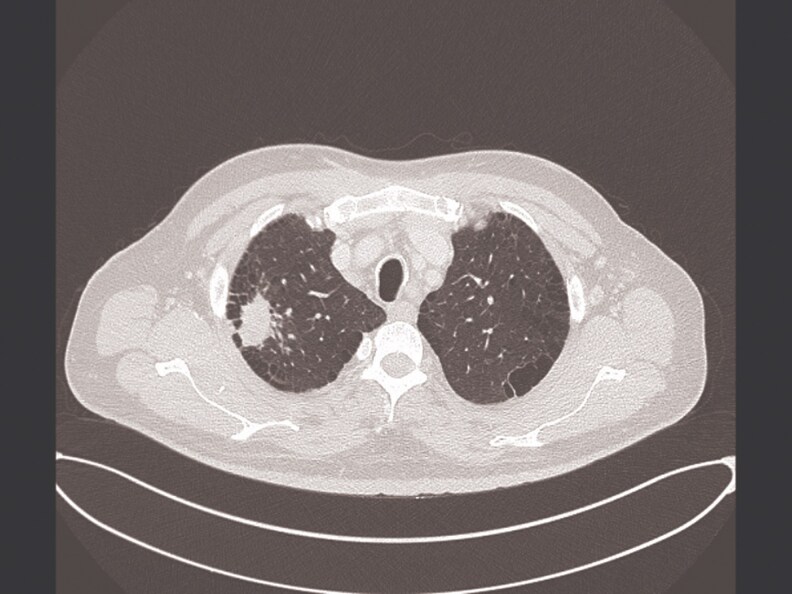
Radiological image (eg, CT scan) showing a subpleural nodule with satellite nodules mimicking lung cancer in a pulmonary *M riyadhense* infection (case 6; Table 2).

**Figure 7.**
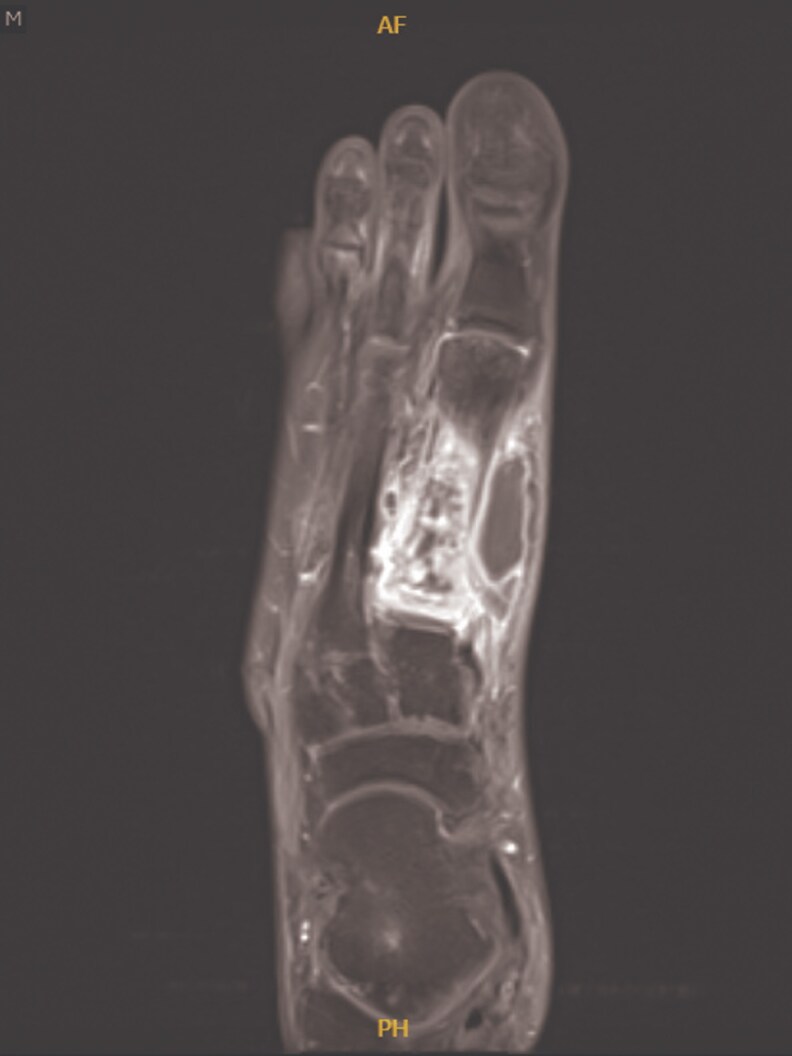
Imaging (eg, MRI or X-ray) depicting a metatarsal lesion associated with osteomyelitis in a *M riyadhense* infection (case 7; Table 2).

**Figure 8.**
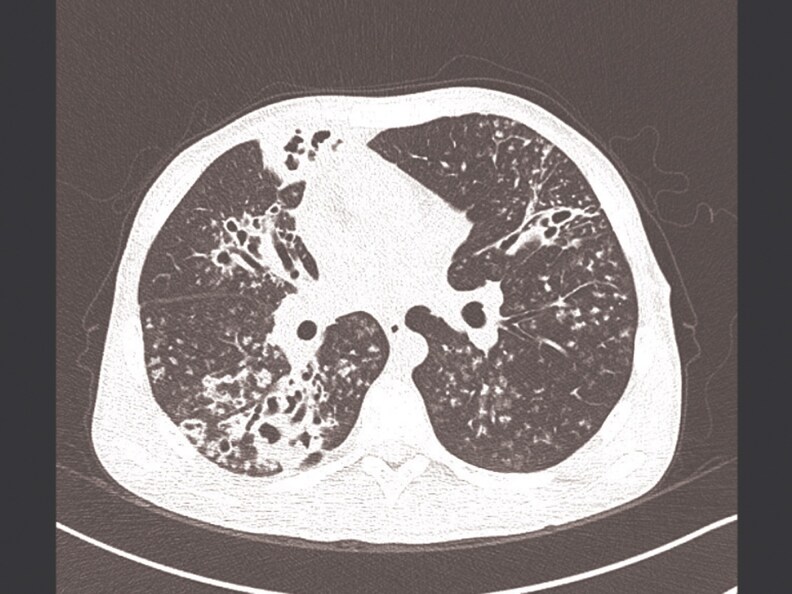
Radiological image (eg, CT scan) showing cavitary lesions in a pulmonary *M riyadhense* infection and possibly histological findings from a renal biopsy confirming immune complex glomerulonephritis (case 8; Table 2).

Pulmonary infections dominated (22 in 32, 68.75%), presenting with chronic cough (18 in 22, 81.8%), fever (14 in 22, 63.6%), night sweats (12 in 22, 54.5%), weight loss (11 in 22, 50%), and hemoptysis (4 in 22, 18.2%) (Tables 1 and 2). Radiological findings included cavitary lesions (16 in 32, 50%), consolidation (11 in 32, 34.38%), and lymphadenopathy (8 in 32, 25%), as seen in Figures 2–5 and 8. Five of the 8 new cases had pulmonary involvement (cases 2–6 and 8), with case 6 mimicking lung cancer due to a subpleural nodule with satellite nodules (Figure 6). Extrapulmonary infections (10 in 32, 31.25%) included lymphadenitis (4 cases; eg, Figure 1), osteomyelitis (2 cases; eg, Figure 7), cranium/spine infections (2 cases), maxillary sinusitis (1 case), and a novel immune complex glomerulonephritis (case 8; Figure 8). Disseminated disease occurred in 5 cases (15.6%), predominantly in patients who are immunosuppressed (4 in 12 vs 1 in 20 in immunocompetent; *P* = .03), with lymphadenopathy as a common secondary site (cases 2, 6, and 7; Tables 1 and 2). Case 8's glomerulonephritis, marked by acute kidney injury and proteinuria, represents the first reported renal complication of *M riyadhense*, mirroring TB-associated glomerulonephritis (Table 2) [12]. Symptom duration before presentation ranged from 2 weeks to 4 months (median 2.5 months), with longer delays in extrapulmonary cases (median 3 months vs 2 months for pulmonary; *P* = .06).

### Diagnosis

Diagnosis was challenging due to *M riyadhense*'s resemblance to *M tuberculosis*. Acid-fast bacilli (AFB) staining was positive in 18 in 32 cases (56.25%), but TB-PCR was negative in all 28 tested cases, prompting suspicion of NTM (Table 2). Culture confirmation, using Middlebrook agar or MGIT, took 3–7 weeks (median 6 weeks), contributing to diagnostic delays (eg, 8 weeks in cases 2, 4, and 6; Table 2). Molecular sequencing (16S rRNA in 25 cases, hsp65 in 10, rpoB or ITS in 5) was required for definitive diagnosis in all cases, as commercial line-probe assays misidentified *M riyadhense* as *M tuberculosis* in 6 in 10 tested cases (Tables 1 and 2) [1–3]. Histopathology, performed in 15 cases, showed granulomatous inflammation (12 in 15, 80%), with necrosis in 8 in 15 (53.3%), but was nonspecific without molecular confirmation (eg, case 4; Table 2). Diagnostic methods varied: sputum culture was used in 18 pulmonary cases, bronchoalveolar lavage (BAL) in 5, biopsy in 7 extrapulmonary cases, and endobronchial ultrasound (EBUS) in 1 (case 6; Figure 6). The novel glomerulonephritis case (case 8) required renal biopsy to confirm immune complex deposition, highlighting diagnostic complexity (Table 2; Figure 8). MALDI-TOF MS, recently updated to include *M riyadhense*, was used in 2 cases but remains limited by database availability [14].

### Treatment Outcomes

Initial treatment often involved standard anti-TB regimens (isoniazid, rifampin, ethambutol, pyrazinamide) in 15 in 32 cases (46.9%) due to suspected TB, but modifications were required in 12 cases (80%) due to adverse effects (eg, hepatotoxicity in cases 3 and 7; Table 2), clinical relapse (case 2), or microbiological confirmation (cases 4 and 8; Tables 1 and 2). Macrolide-based regimens (clarithromycin or azithromycin) combined with rifampin, ethambutol, or fluoroquinolones (moxifloxacin) were used in 20 in 32 cases (62.5%) and associated with cure or improvement in 28 in 32 cases (87.5%) (Tables 1 and 2). Treatment duration ranged from 9 to 15 months (median 12 months), with extrapulmonary and disseminated cases requiring longer therapy (median 14 months vs 10 months for pulmonary; *P* = .02; eg, case 7, Table 2). Surgical interventions, including debridement (cases 7 and 8) and biopsy (cases 1 and 4), were critical in 5 in 32 cases (15.6%) (Table 2). Susceptibility testing (Table 3) showed 100% susceptibility to rifampin, ethambutol, azithromycin, moxifloxacin, and rifabutin, and 93% to clarithromycin, guiding therapy adjustments (eg, case 1 switched from clarithromycin to moxifloxacin due to gastrointestinal effects; Table 2). Relapse occurred in 2 in 32 cases (6.25%), both associated with inadequate initial regimens (Table 1, case 3; Table 2, case 2). Follow-up imaging confirmed resolution in 12 in 15 pulmonary cases (eg, Figures 3*B*, 4*B*, and 5*B*), with residual cavities in 3 cases (Table 2). The glomerulonephritis case (case 8) improved with steroids and azithromycin-based therapy, with normalized renal function at 3 months (Table 2; Figure 8).

**Table 3.** Antimicrobial Susceptibility Profiles of *M riyadhense* Isolates

Drug	Previous Cases (Case No.)	New Cases (Case No.)	Total Tested	% Susceptible
Rifampin	S (1,2,3,4,6,7,21,22,23,24)	S (1,2,3,4,6,7,8)	17	100%
Clarithromycin	S (1,2,3,4,6,21,22,23,24), I (7)	S (1,3,4,6,7,8)	16	93%
Ethambutol	S (1,2,3,4,6,7,23,24)	S (1,2)	11	100%
Ciprofloxacin	S (1,2,3,6), I (4,23)	S (2)	7	71%
Amikacin	R (1), S (2,3,4,6)	S (2)	6	83%
Isoniazid	I (1), S (2,3,23), R (6,7)	-	6	50%
Azithromycin	-	S (3,4,6,7,8)	5	100%
Doxycycline	R (2,3), S (4), I (6,23)	-	5	20%
Streptomycin	S (1,2,3,6,7)	-	5	100%
Moxifloxacin	S (2,3,4)	S (2)	4	100%
Rifabutin	S (1,2,3)	S (2)	4	100%
Co-trimoxazole	S (4), R (23)	S (2)	3	N/A
Ethionamide	S (2,3,6)	-	3	N/A
Linezolid	S (2,3)	S (2)	3	N/A
Cycloserine	S (1,6)	-	2	N/A
P-Aminosalicylate	R (1,6)	-	2	N/A
Pyrazinamide	S (23), R (7)	-	2	N/A
Capreomycin	S (6)	-	1	N/A
Clofazimine	S (1)	-	1	N/A
Imipenem	R (6)	-	1	N/A
Kanamycin	R (6)	-	1	N/A
Levofloxacin	-	-	1	N/A
Prothionamide	S (1)	-	1	N/A

Abbreviations: S, susceptible; R, resistant; I, intermediate; N/A, not applicable.

## DISCUSSION

*M riyadhense* presents unique diagnostic and therapeutic challenges due to its clinical, radiological, and immunological overlap with *M tuberculosis* and other NTMs, such as MAC and *M abscessus* [1, 11]. The predominance of cases in Saudi Arabia (22 in 32, 68.75%; Tables 1 and 2) suggests a regional environmental reservoir, possibly linked to soil or water, as seen with other NTMs [7, 10]. The global spread to France, Bahrain, South Korea, and the UAE (Table 1) indicates a broader public health concern, potentially driven by migration or travel, warranting surveillance in nonendemic regions [2, 3, 9]. The balanced distribution between patients who are immunocompetent (62.5%) and immunosuppressed (37.5%) contrasts with MAC, which predominantly affects hosts who are immunocompromised, but aligns with *M kansasii*'s broader host range, suggesting *M riyadhense*'s adaptability to diverse immune environments (Tables 1 and 2) [8].

The pulmonary predominance (68.75%) and symptoms mimicking TB (eg, cough, fever; Tables 1 and 2) led to frequent misdiagnosis, with 15 in 32 cases initially treated for TB (Tables 1 and 2). Radiological findings, such as cavitary lesions (50%) and consolidation (34.38%; Figures 2–5 and 8), further complicated differentiation, as seen in case 6's cancer mimic (Figure 6). Extrapulmonary manifestations (31.25%), including lymphadenitis (Figure 1), osteomyelitis (Figure 7), and glomerulonephritis (Figure 8), highlight *M riyadhense*'s multisystem potential, particularly in patients who are immunosuppressed (4 in 5 disseminated cases; Tables 1 and 2). The novel glomerulonephritis case (case 8; Table 2) parallels TB-associated renal complications, suggesting shared antigenic triggers or immune complex deposition mechanisms, which merit further immunopathological studies [12].

Diagnosis remains a critical bottleneck. AFB staining's low sensitivity (56.25%) and line-probe assay failures (6 in 10 misidentifications; Tables 1 and 2) underscore the necessity of molecular sequencing (16S rRNA, hsp65), which was pivotal in all 32 cases [1–3]. The median 6-week diagnostic delay (Table 2) reflects slow culture growth and limited access to advanced diagnostics in some settings, risking inappropriate therapy and prolonged morbidity. The inclusion of *M riyadhense* in updated MALDI-TOF MS databases is promising but insufficient without widespread adoption [14]. Developing *M riyadhense*-specific line-probe assays or multiplex PCR panels could streamline diagnosis, particularly in endemic regions.

Treatment outcomes highlight the efficacy of macrolide-based regimens (clarithromycin, azithromycin) combined with rifampin or fluoroquinolones, with 87.5% cure/improvement rates (Tables 1 and 2). The 100% susceptibility to rifampin, ethambutol, and azithromycin (Table 3) supports their use as first-line agents, contrasting with *M abscessus*'s frequent macrolide resistance [8]. Adverse effects, such as hepatotoxicity (cases 3 and 7; Table 2), necessitated regimen changes in 12 in 15 anti-TB-treated cases, emphasizing the need for susceptibility-guided therapy (Table 3). Surgical intervention's role in 15.6% of cases (eg, case 7's debridement; Table 2) aligns with NTM guidelines for refractory infections [9]. The low relapse rate (6.25%) suggests durable responses with tailored therapy, though longer follow-up is needed.

Limitations include the small sample size (32 cases), retrospective design, and lack of *M riyadhense*-specific susceptibility breakpoints, which may overestimate susceptibility to some agents (Table 3). The absence of environmental data limits understanding of transmission. Future research should investigate environmental reservoirs using genomic epidemiology to map transmission, develop rapid, *M riyadhense*-specific diagnostic assays to reduce delays, conduct prospective trials to standardize treatment regimens and durations, and explore host-pathogen interactions, particularly for novel complications such as glomerulonephritis (case 8; Table 2; Figure 8).

Enhanced clinician awareness, especially in endemic regions, and investment in molecular diagnostics are critical to improve outcomes, reduce misdiagnosis, and mitigate the public health impact of *M riyadhense*.

## CONCLUSIONS

*M riyadhense* has emerged as a significant NTM, particularly in Saudi Arabia, where it appears endemic. Although it typically presents with pulmonary manifestations closely resembling TB, extrapulmonary involvement—including osteomyelitis, lymphadenitis, and, as demonstrated in this report, immune complex–mediated glomerulonephritis—highlights its potential for multisystem involvement. Accurate diagnosis often requires molecular methods, such as sequencing of the 16S rRNA, hsp65, rpoB, or ITS genes, due to the frequent misidentification as *M tuberculosis*. Recommended treatment includes macrolides (clarithromycin or azithromycin), rifampin, ethambutol, and occasionally fluoroquinolones, with treatment durations ranging from 9 to 15 months, depending on disease severity and location. Despite growing awareness, underreporting of *M riyadhense* persists, underscoring the need for heightened clinical vigilance, standardized diagnostic protocols, expanded susceptibility testing, and the development of more accessible diagnostic tools to improve outcomes.
